# Outdoor, Indoor, and Personal Exposure to VOCs in Children

**DOI:** 10.1289/ehp.7107

**Published:** 2004-07-15

**Authors:** John L. Adgate, Timothy R. Church, Andrew D. Ryan, Gurumurthy Ramachandran, Ann L. Fredrickson, Thomas H. Stock, Maria T. Morandi, Ken Sexton

**Affiliations:** ^1^Division of Environmental Health Sciences, School of Public Health, University of Minnesota, Minneapolis, Minnesota, USA;; ^2^University of Texas, Houston Health Science Center, School of Public Health, Houston, Texas, USA;; ^3^University of Texas, School of Public Health, Brownsville Regional Campus, Brownsville, Texas, USA

**Keywords:** air pollution, elementary school children, ethnicity, health risk, race, SHIELD study

## Abstract

We measured volatile organic compound (VOC) exposures in multiple locations for a diverse population of children who attended two inner-city schools in Minneapolis, Minnesota. Fifteen common VOCs were measured at four locations: outdoors (O), indoors at school (S), indoors at home (H), and in personal samples (P). Concentrations of most VOCs followed the general pattern O ≈ S < P ≤ H across the measured microenvironments. The S and O environments had the smallest and H the largest influence on personal exposure to most compounds. A time-weighted model of P exposure using all measured microenvironments and time–activity data provided little additional explanatory power beyond that provided by using the H measurement alone. Although H and P concentrations of most VOCs measured in this study were similar to or lower than levels measured in recent personal monitoring studies of adults and children in the United States, *p*-dichlorobenzene was the notable exception to this pattern, with upper-bound exposures more than 100 times greater than those found in other studies of children. Median and upper-bound H and P exposures were well above health benchmarks for several compounds, so outdoor measurements likely underestimate long-term health risks from children’s exposure to these compounds.

Although ambient levels of the major criteria pollutants have declined in the United States over the last 30 years, much less is known about exposure to many of the 189 hazardous air pollutants identified in the 1990 Clean Air Act Amendments (Clean Air Act Amendments 1990). Volatile organic compounds (VOCs) are an important class of outdoor air toxics because they are ubiquitous and associated with increased long-term health risks (Pratt et al. 2000; Woodruff et al. 1998). VOCs are also an indoor air quality issue because humans spend, on average, nearly 90% of their time indoors (Klepeis et al. 2001). VOCs in ambient air largely originate from mobile and industrial sources. The cumulative risk from exposure to multiple VOCs and other air pollutants is not known, and limited evidence suggests that the minority populations residing in inner-city neighborhoods have disproportionately higher exposures (Kinney et al. 2002; Metzger et al. 1995). There are relatively few data on VOC exposures in children or for minority populations in the United States (Adgate et al. 2004).

Past research has shown that VOCs are typically higher indoors than outdoors and that construction materials and building characteristics, such as the presence of an attached garage or air exchange rate, can influence levels or indoor:outdoor ratios (Levin 1989; Otson et al. 1994; Wallace 2001; Wallace et al. 1985). The contribution of indoor sources, such as consumer products and environmental tobacco smoke (ETS), is the largest source of variability in measured personal and indoor levels of many compounds (Sexton et al. 2004a; Wallace 2001). Compounds associated with consumer product use include *p*-dichlorobenzene (moth cakes, room air fresheners, toilet bowl deodorizers), chloroform (chlorinated water), and the fragrances α- and β-pinene and *d*-limonene (cleaning products, room fresheners) (Wallace 1991a, 1991b). Benzene and styrene have been shown to be elevated in homes with smokers, but these compounds also originate from traffic and are often higher in urban areas (Edwards et al. 2001).

Characterizing air pollution exposures in inner-city children is important for providing benchmarks for assessing environmental justice, estimating health risks, recommending interventions, and designing epidemiologic studies. We measured outdoor, indoor at school and home, and personal VOC concentrations for an ethnically and racially diverse sample of inner-city children in Minneapolis, Minnesota. These children were participants in the School Health Initiative: Environment, Learning, and Disease (SHIELD) study (Sexton et al. 2000, 2003), which selected participants at random with known probabilities from a defined sampling frame so inferences could be drawn about sociodemographic groups in two elementary schools. This analysis examines the distribution of exposures to common VOCs measured in the personal air and three primary microenvironments where these children spent time. We also examine how VOC exposures vary by sociodemographics, source/housing characteristics, and time–activity patterns and compare these results with health benchmarks and levels observed in recent VOC exposure studies in children and adults.

## Materials and Methods

The SHIELD study was approved the University of Minnesota Research Subjects’ Protection Program Institutional Review Board: Human Subjects Committee and examined children’s exposures to a complex mixture of environmental agents, including VOCs and other chemical and biological agents. A detailed description of the SHIELD study design, eligibility criteria, sample selection, informed consent process, and response rates has been published (Sexton et al. 2000, 2003) and is briefly summarized here.

Children from two inner-city schools serving predominantly low-income households (> 90% qualified for free or reduced-price meals in the National School Lunch/Breakfast Program) in Minneapolis were recruited between November 1999 and January 2000. The three largest racial/ethnic groups that enrolled in SHIELD were African Americans, Hispanics, and Somalis, with a smaller number of Caucasians, Native Americans, Southeast Asians, and those declaring “other” or mixed-race ancestry. We used a stratified random sample to ensure an adequate number of subjects within the following defined subgroups of children: school (Lyndale, Whittier), grade (2nd, 3rd, 4th, 5th), language (English or non-English language spoken at home), and sex (female, male). This produced 32 distinct strata, with a target of five “index” children per stratum, for a target sample size of 80 children/school. A total of 153 index children were recruited for the study and eligible for VOC monitoring.

VOC measurements were obtained in winter (24 January–18 February) and spring (9 April–12 May) 2000. Fifteen VOCs were monitored concurrently in four locations using organic vapor monitors (OVMs; model 3520; 3M Corporation, St. Paul, MN): in the personal breathing zone (P), indoors in the child’s primary residence (H), indoors in five randomly selected classrooms in each school (S), and outdoors (O) at each school. To ensure a relatively high percentage of detectable concentrations, the duration of VOC sampling varied by location, based on logistical considerations and VOC concentrations measured during a pilot study: *a*) P and H measurements were collected continuously for 2 days (~ 48 hr), *b*) S measurements were collected each school day by capping the OVMs after school hours (average weekly sampling duration ~ 31 hr over 5 days), and *c*) O measurements were collected at each school continuously from Monday morning to Friday afternoon each week (~ 103 hr).

The P and H samplers were deployed simultaneously during a household visit on a Sunday, Monday, Tuesday, or Wednesday evening by SHIELD field teams. The P samplers were attached to the clothes in the breathing zone of the child, and the H sampler was placed in the room where the child spent the most time while awake. At night subjects were instructed to place the P monitor beside their bed. During the monitoring period each subject kept a time–activity diary (TAD), recording time the child spent in seven primary microenvironments (inside at home, school, and other; outside at home, school, and other; and in transit) as well as data on exposure to ETS and other potential exposure modifiers (e.g., use of cleaning products) and the number of hours that doors and windows were open. On the second day after deploying the P/H OVMs, study staff collected the samplers and checked the TAD for completeness. The SHIELD baseline questionnaire provided data on housing type, some source-related characteristics, and other sociodemographic information.

The OVMs are charcoal-based passive air samplers. The precision, accuracy, and suitability of these VOC badges for outdoor, indoor, and personal sampling have been demonstrated in previous studies (Chung et al. 1999a, 1999b; Stock et al. 1999). Target VOCs were extracted from OVMs using a 2:1 (vol/vol) mix of double-distilled acetone and carbon disulfide (Sigma-Aldrich, St. Louis, MO), which provided a very low background for target analytes. All extracts were analyzed by gas chromatograph/mass spectrometer with a Hewlett-Packard (HP) 5890 Series II Plus gas chromatograph with an HP 5972 mass spectrometer detector, HP 18593B autosampler, Vectra 486 computer with EnvironQuant ChemStation software, and NBS75K Spectra Library (all from Hewlett Packard, Palo Alto, CA), using an RTX-1/60 m/0.25 μm inner diameter/1 mm film thickness capillary column. Approximately 10% of H and S samples collected during the study were duplicates: Correlation coefficients (*R*^2^) for measurable values were > 0.90 for most VOCs, with lower values observed for β-pinene (0.85), benzene (0.84), and methylene chloride (0.59). The percent median relative absolute difference (the median of the ratios of within-pair absolute differences divided by the within-pair mean, multiplied by 100) for duplicate samples was 9% and ranged from 6% (β-pinene) to 14% (methylene chloride).

Statistical analyses were performed using SAS (version 8.0; SAS Institute Inc., Cary, NC) and S-plus (version 6.1; Insightful Corp., Seattle, WA) using log-transformed data because most VOC measurements were right skewed. Concentrations that were less than the analytical detection limit but produced an instrument reading > 0 were included in calculations. Concentrations that produced an instrument reading ≤ 0 (typically due to blank subtraction) were also included in calculations by assigning them a value of one-half the analytical detection limit. This substitution was infrequently done and had little effect on the P and H results: Among the 120 sample sets reported here (15 chemicals × 2 seasons × 4 sampling locations), more than half had no ≤ 0 instrument readings, and 85% had ≤ 0 instrument readings occurring < 10% of the time. The seven sample sets with a high proportion (> 50%) of ≤ 0 instrument readings were either O or S measurements of chloroform, *d*-limonene, β-pinene, or styrene.

**Figure 1:**
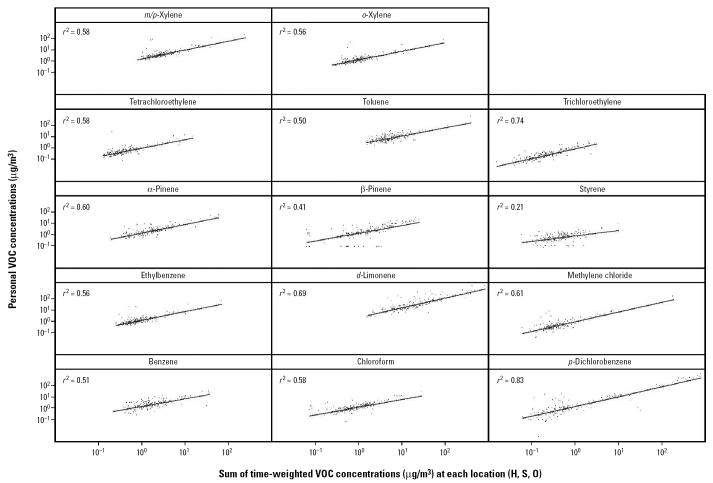
Scatter plot of correlation between a 48-hr P VOC exposure and a time-weighted average of VOC exposure from major microenvironments where children spent time each day.

**Figure 2:**
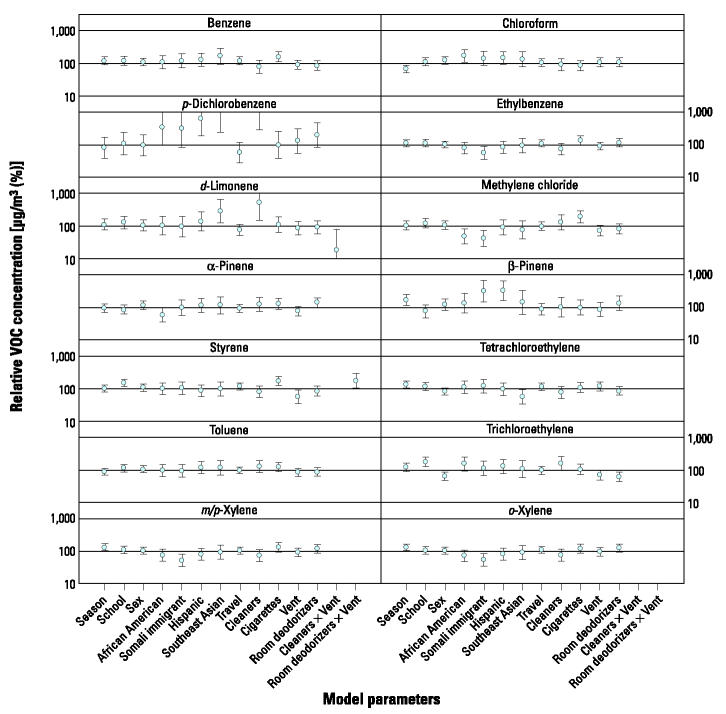
Age-adjusted regression results showing variability (95% CIs) in P VOC concentrations for study design (e.g., school, demographic categories), source (e.g., use of cleaning products), or exposure modification (e.g., ventilation) variables. Vent, ventilation. Interaction terms are displayed only if statistically significant: season, winter 2000 vs. spring 2000; school, Whittier vs. Lyndale; sex, female vs. male; African American, Somali immigrant, Hispanic, and Southeast Asian vs. other (including Caucasian); travel, > 1.5 hr on highway or road today; cleaners, > 0 hr spent using cleaning supplies today; cigarettes, > 0 cigarettes smoked in your presence today; Vent, > 12 hr doors and windows were left open for ventilation today; room deodorizers; cleaners × Vent; room deodorizers × Vent.

**Figure 3:**
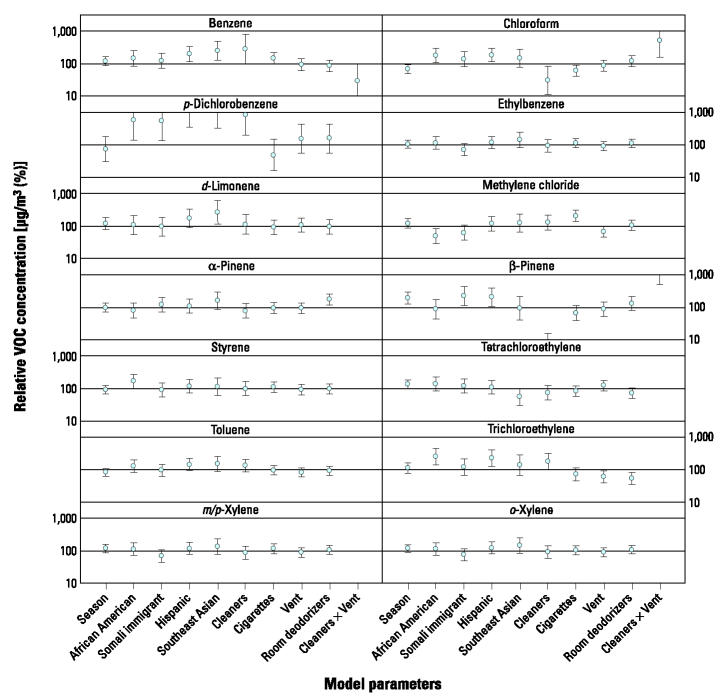
Age-adjusted regression results showing variability (95% CIs) in H VOC concentrations for study design (e.g., demographic categories), source (e.g., use of cleaning products), or exposure modification (e.g., ventilation) variables. Vent, ventilation. Interaction terms are displayed only if statistically significant: season, winter 2000 vs. spring 2000; African American, Somali immigrant, Hispanic, and Southeast Asian vs. other (including white); cleaners, > 0 hr spent using cleaning supplies today; cigarettes, > 0 cigarettes smoked in your presence today; Vent, > 12 hr doors and windows left open for ventilation today; room deodorizers; cleaners × Vent.

To estimate the contribution of the indoor and outdoor microenvironments to P exposure, we examined the time-weighted model


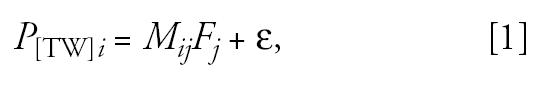


where *i* is the index of chemical and the *j* the index of microenvironment *M* (H, S, or O), *F* is the fraction of time spent in microenvironment *j*, and ɛ is the error term (Figure 1). To examine the potential influence of school or sociodemographics (study design variables) and VOC emitters (sources) and modifiers (e.g., ventilation) of personal exposure across the population, we also conducted weighted linear regression using the model


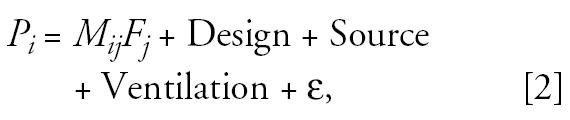


where Design (season, school, English or non-English-speaking home, race/ethnicity, and grade), Source (source variables, e.g., presence of a smoker in household), and Ventilation [high (> 12 hr windows/doors open) vs. low ventilation] were included as covariates. Variables included in the regression were selected based on associations observed in previous studies and cut points for comparison groups based on continuous variables (e.g., high or low average hours of ventilation) constructed to obtain groups of approximately equal sizes. We also tested two-way interactions between season, source variables, and ventilation but report only significant associations in subsequent figures. Results for carbon tetrachloride are not included in Figures 2 and 3 because it is no longer produced, and measured levels represent global background (Sexton et al. 2004b).

## Results

We obtained 181 matched P and H measurements (*n* = 93 winter, *n* = 88 spring) and a completed TAD questionnaire from 113 subjects (68 subjects in both seasons, 25 winter only, and 20 spring only), for a weighted response rate of 84.5% in winter and 73.6% in spring. The sociodemographics and household characteristics of the children who provided these samples are summarized in Table 1. The subjects who volunteered for VOC sampling were evenly distributed across grades and schools; were predominantly African American, Hispanic, or Somali; and were slightly more likely to be male. Even though the number of non-English-speaking participants was substantially larger than the number of English-speaking participants (*n* = 76 vs. *n* = 37), the former group represented only about half (52%) of the weighted sample due to adjustment for oversampling in the non-English-speaking strata. More than one-third of the children (35.6%) were born outside the United States, and nearly 22% lived in households where at least one person smoked, although this factor was reported to be more common in African-American households than for other races/ethnicities. Most of the families lived in rented apartments and did not have an attached garage or central air conditioning. Three-quarters of the families reported using room deodorizers. The children who provided VOC samples were between 7 and 13 years of age, with a mean age of 9.1 ± 1.4 years.

Weighted summary statistics that estimate the median and variability in VOC concentrations for the O, S, H, and P samples are presented in Table 2. Six VOCs (benzene, carbon tetrachloride, ethylbenzene, toluene, *o*-xylene, and *m*/*p*-xylene) were detected in all O samples. All O VOC concentrations were low compared with other measurement locations but displayed some seasonal variability, with five compounds (chloroform, *p*-dichlorobenzene, *d*-limonene, α-pinene, and trichloroethylene) more frequently detectable and at higher concentrations in spring compared with winter. Benzene, carbon tetrachloride, ethylbenzene, tetrachloroethylene, trichloroethylene, toluene, and the xylenes were present in more than half of the O, S, H, and P samples, but methylene chloride was above the detection limit in < 25% of samples from all measurement locations. Concentrations of β-pinene and styrene did vary substantially by measurement location and were more frequently detectable in H and P samples compared with S and O samples. Excluding trichloroethylene and carbon tetrachloride (because their concentrations were uniform across all microenvironments) and methylene chloride (because of low percent detectable), the relative concentration of VOCs at each location was either S < O < P ≤ H (benzene, *d*-limonene, and *p*-dichlorobenzene) or O ≈ S < P ≤ H (all other compounds). Across both seasons, the ratios of P:O and P:S medians were much greater than 1 for all compounds; the ratios of P:H medians ranged from 0.6 to 0.9 for all compounds except *p*-dichlorobenzene (1.4); and the ratios of S:O medians were > 1 for chloroform, *p*-dichlorobenzene, and *d*-limonene, and ≤ 1 for the other compounds. The within-measurement location variability (90th:50th percentile ratio) was relatively small for O samples (range, 1.0–3.5) and S samples (range, 1.0–4.2). For P samples, the 90th:50th percentile ratio ranged from 2.2 to 6.8 for all compounds except *p*-dichlorobenzene, which was 167 in winter and 67 in spring, indicating substantial between-child variability for this compound. This pattern is also clear in the H samples: The 90th:50th percentile ratio values ranged from 2.1 to 8.5 for all compounds except *p*-dichlorobenzene, which was > 475 in both seasons, indicating substantial between-residence variability for this compound.

Time–activity patterns for these children indicate that they spent an average of 25% of their time at school and > 90% of their time indoors at any location (Table 3). On average, SHIELD study children spent slightly more time in a vehicle than they did outdoors at any location, but time outdoors or in vehicles averaged < 8% of the day. Although an adult smoker was reported present in about one in five households, slightly more than one-quarter of the children reported some exposure to ETS, with the 90th percentile for minutes of ETS exposure being slightly less than 1 hr per day.

To examine the contribution of H, S, and O microenvironments to personal exposure, we plotted P versus time-weighted exposures estimated using concentrations and time–activity data for each microenvironment (Equation 1, Figure 1). The combination of H, S, and O microenvironments explain > 50% of the observed variability for all compounds except styrene (0.21) and β-pinene (0.41). As shown in Table 4, P exposure was dominated by the contribution from the residential environment for all compounds: The time-weighted model with H, S, and O measurements explained little more variability in personal exposure than did the model with the H measurement alone.

Regression results for P exposures in Figure 2 show 95% confidence intervals (CIs) for the variability in age-adjusted VOC concentrations for various population subgroups compared with a referent group (results not including the 100% line represent a statistically significant departure from geometric mean values). Variability in P exposure was not associated with sex, time spent in travel, residence ventilation, or use of room deodorizers. Adult tobacco use was associated with elevated styrene and benzene personal exposures, and use of cleaning supplies was associated with higher *d*-limonene, *p*-dichlorobenzene, and trichloroethylene P exposures. Variation among racial/ethnic groups was explored by comparing each major group sampled with the Other group, which consisted of Caucasians, Native Americans, and those of mixed race/ethnicity. Compared with this group, African Americans had higher chloroform exposures, Hispanics had higher *p*-dichlorobenzene and β-pinene exposures, Somalis had higher β-pinene exposures, and Southeast Asians had higher *d*-limonene and *p*-dichlorobenzene exposures. Compared with the referent group, Somalis had lower ethylbenzene, methylene chloride, and xylene exposures, and African Americans had lower methylene chloride and α-pinene exposures. Significant associations were observed for the interaction between ventilation and household cleaner use (lower *d*-limonene) and ventilation and room deodorizers (higher styrene).

Regression results for H exposures in Figure 3 display both divergence and some consistency with the trends observed for P exposures. In divergence with the P results, adult tobacco use was not associated with elevated indoor styrene or benzene levels in SHIELD residences. Although the use of cleaning supplies was still associated with higher *p*-dichlorobenzene levels, it was also associated with lower β-pinene and chloroform levels. Room deodorizer use was associated with higher indoor α-pinene but lower trichloroethylene levels. African-American households had higher chloroform, *p*-dichlorobenzene, and trichloroethylene levels relative to referent households. Exposures to air freshener and fragrance compounds still varied substantially among immigrant subgroups but showed consistency with P results: Higher concentrations of *d*-limonene (Southeast Asians) and *p*-dichlorobenzene (Hispanics and Southeast Asians) were observed for some households, and lower levels of the xylenes were observed in Somali residences. Significant associations were observed for the interaction between ventilation and household cleaner use (elevated chloroform but decreased benzene levels). Dry cleaning use was infrequently reported in this population, and therefore this exposure factor was not included in the regression models, although tetrachloroethylene levels were lower in households with greater ventilation.

## Discussion

In this study we obtained concurrent outdoor, school and home indoor, and personal VOC samples from a diverse population of inner-city school children. The suite of VOCs reported here are compounds with long-term health risks and include *a*) those that are primarily from indoor sources (e.g., chloroform, *p*-dichlorobenzene, *d*-limonene, α- and β-pinene), *b*) those that have indoor and outdoor sources (e.g., benzene, ethylbenzene, styrene, toluene, tetrachloroethylene, *m*/*p*-and *o*-xylene), and *c*) those that originate mainly from outdoor air (e.g., carbon tetrachloride). For compounds detected in > 70% of samples, both median and 90th percentile concentrations followed the general pattern O ≈ S < P ≤ H or S < O < P ≤ H across the measured microenvironments. The only exceptions to this were carbon tetrachloride, which had similar concentrations in every measured microenvironment because measured levels represent global background, and trichloroethylene, for unknown reasons. It is clear from our analysis that the H microenvironment was the largest and that the O and S microenvironments were relatively small contributors to children’s personal exposure to these hazardous air pollutants.

The O VOC levels measured in Minneapolis for this study are relatively low compared with those in other large metropolitan areas in the United States (Sexton et al. 2004b), primarily because the Twin Cities metropolitan area is downwind of rural areas in the United States and Canada that have low VOC emissions, have relatively infrequent atmospheric inversions, and have no physical barriers that trap pollutants. The 2- to 5-day sampling durations used in this study allowed for enough material to be collected so that the percentage of samples above the detection limit was reasonably good for most compounds: Only methylene chloride, α- and β-pinene, and styrene were found in less than half of the O and S samples in both seasons, and all compounds except methylene chloride were found in > 70% of H and P samples.

Indoor VOC concentrations are a function of both outdoor sources (e.g., vehicle exhaust) and indoor sources (e.g., ETS, consumer products). Previous population-based studies in the United States suggest that levels of many VOCs are typically higher inside residences than in matched outdoor concentrations (Sexton et al. 2004b; Wallace 2001) because the source strength of indoor emissions is a stronger influence than the infiltration of outdoor air for many of these pollutants, especially those associated with fragrances and other consumer products (Kim et al. 2001). There was considerable variability in exposure to ETS between racial/ethnic groups: Time-diary and biomarker data indicate that African-American children in SHIELD had higher tobacco exposures than did other racial/ethnic groups (Sexton et al. 2004a). In all cases the indoor home environment had higher concentrations than did indoor air at these schools, possibly because of greater air exchange in the schools, but also because there are fewer strong sources in schools than exist in the residential environment. Children’s P concentrations were slightly lower than H for all compounds except *p*-dichlorobenzene. Research in adults indicates that P concentrations tend to be higher than matched residential concentrations, which are higher than matched outdoor levels, although in some communities with higher ambient concentrations, indoor and outdoor levels may be nearly the same (Kim et al. 2001; Kinney et al. 2002; Sexton et al. 2004b; Wallace et al. 1985).

To put our results in context, we compared them with three recent studies that measured personal and indoor VOC exposures in the central northern United States: *a*) nonsmoking Minneapolis–St. Paul adults who participated in the Hazardous Air Pollution Study (HAPS) (Sexton et al. 2004b, 2004c); *b*) midwestern U.S. (OH, IL, IN, MI, WI, and MN) adults (*n* = 258 > 21 years old) and children (*n* = 55 < 14 years old) who participated in the National Human Exposure Assessment Survey (NHEXAS) Region V study (Pellizzari et al. 1999); and *c*) 3- to 13-year-old participants (*n* = 73) who provided VOC measurements in the Minnesota Children’s Pesticide Exposure Study (MNCPES) (Adgate et al. 2004). All three studies used OVMs to measure VOCs, although a slightly different suite of compounds was measured in MNCPES and NHEXAS, whereas the same compounds were measured in the HAPS and SHIELD studies.

Median and upper-bound exposures in SHIELD were similar to or lower than those experienced by HAPS adults and NHEXAS Region V adults/children for all VOCs except *p*-dichlorobenzene. This difference likely occurs because children are in fewer “high-exposure” microenvironments outside the home, such as vehicles in traffic. With *p*-dichlorobenzene the only exception, median and upper-bound exposures in SHIELD were lower than those experienced by children in the MNCPES study. The MNCPES VOC samples were collected from a probability sample of urban, suburban, and rural Minnesota households with children who were similar in age to the SHIELD population. The main differences between the two studies were that the MNCPES samples were collected during the summer, and the MNCPES households were > 90% nonminority and had substantially higher household incomes (median > $50,000) than did the households that participated in SHIELD (median < $20,000) (Adgate et al. 2000; Sexton et al. 2003). In our judgment, the effect of season is unlikely to explain the observed differences, which are more likely related to differences in housing stock and indoor source use between these two study populations.

It is notable, however, that *p*-dichlorobenzene exposures were substantially higher in SHIELD P and H samples than in the other three studies, especially at the upper end of the exposure distribution. For example, SHIELD median P exposures to *p*-dichlorobenzene were 1.6–2.9 times higher than in the other three studies, with 90th percentile concentrations ranging from 17 to 30 times higher in SHIELD. This difference is even more striking for H concentrations: SHIELD median concentrations were 2- to 4-fold higher than in the HAPS, NHEXAS, and MNCPES, with 90th percentile concentrations ranging from 33 times higher to more than 250 times higher in SHIELD than in the other three studies. The distribution of exposure to *p*-dichlorobenzene in SHIELD children is bimodal, with 25 individuals with 48-hr average mean P exposures > 24 μg/m^3^ (or a time-weighted exposure of > 33 μg/m^3^). These high-exposure subjects were somewhat more likely to be male (16 of 25) and were predominantly Hispanic (13 of 25) and African American (6 of 25), and a large proportion (14 of 25) were in the 4th grade. This association with grade level seems due to chance, because there is no clear grouping of the data within classroom or across time. The primary sources of *p*-dichlorobenzene indoors are consumer products, such as toilet bowl blocks, room deodorizers, and moth cakes (Wallace 1991a, 1991b). Higher *p*-dichlorobenzene levels were not associated with questionnaire responses on frequency of room deodorizer use: Reported use rates in the 25 high-exposure households were similar to reported rates in the remaining study residences.

Overall, children’s time–activity patterns in SHIELD do not vary substantially from those observed in MNCPES and other studies of children that show that children spend 80–90% of their time indoors [U.S. Environmental Protection Agency (EPA) 2002]. Although it is possible to explain a large proportion of the variability in personal exposure for compounds found primarily in the home (e.g., *p*-dichlorobenzene, *d*-limonene), neither residential measurements nor our time-weighted model explains more than half the observed variability in exposure to compounds with both indoor and outdoor sources (e.g., benzene). Although some “high-exposure” microenvironments, such as inside vehicles, are important sources of variability in personal VOC exposure (Weisel et al. 1992), residential concentrations appear to be an important source of variability as well. It is likely that VOC levels are higher when people are at home and using sources, and that average levels obtained using OVMs do not adequately capture the peak levels that presumably exist during these periods of source use. Nonetheless, our data suggest that measuring VOCs in the home environment may be a reasonable proxy for assessing children’s exposure to many of these compounds. Longitudinal studies with repeat measurements over all seasons are needed to confirm the useful approach for estimating children’s VOC exposures in epidemiologic studies (Sexton et al. 2004c).

The main limitation of this work is that we have performed a relatively large number of comparisons, so the results of the regression analysis on the P and H measurements (Figures 2 and 3) should be interpreted with caution. Most of the significant statistical associations we observe appear plausible; for example, the significantly elevated exposures to chloroform, *p*-dichlorobenzene, *d*-limonene, and β-pinene are consistent with existing data on indoor sources, as is the observed relationship between ETS, benzene, and styrene (Wallace 1991a, 1991b). In our judgment, a few of the statistically significant associations we observed are implausible because they lack a clear link to known sources; for example, room deodorizer use was associated with significantly lower trichloroethylene levels in H samples (Figure 3), and cleaning product use was significantly associated with higher carbon tetrachloride levels in H samples (point estimate = 120%; 95% CI, 106–136%). Although we observed elevated exposures to some compounds for the African-American, Hispanic, Somali, and Southeast Asian subpopulations compared with the predominantly Caucasian reference group, the specific exposures are related to known strong indoor VOC sources, so any interventions should be primarily directed at reduction of source use. It is also notable that Somali children had significantly lower exposures to some VOCs associated with vehicle exhaust, such as ethylbenzene. Consistent with this evidence, Somali children also reported somewhat less time spent in transit. Increased ventilation was associated with reduced concentrations of some VOCs with indoors sources (e.g., *d*-limonene) as well as increased concentrations of compounds with outdoor sources (e.g., styrene). Analysis of interactions among source use, ventilation, and season indicated that these three factors together and season alone had no discernable effect on VOC levels.

To put measured values in the context of related health effects, we compared VOC levels in this study with acceptable risk limits for benzene, carbon tetrachloride, chloroform, *p*-dichlorobenzene, methylene chloride, and trichloroethylene. These six VOCs are designated human carcinogens by several regulatory authorities and thus have regulatory guidance for outdoor air levels (Sexton et al. 2004b). The established risk threshold in Minnesota is the airborne concentration (micrograms per cubic meter), which, if breathed over a 70-year lifetime, is estimated (using a 95th percentile upper-bound estimate) to increase an exposed individual’s lifetime cancer risk by 1 × 10^−5^ (Minnesota Pollution Control Agency 2004). All median and 90th percentile concentrations in P, H, S, and O samples were below the acceptable risk level for methylene chloride (20 μg/m^3^) and trichloroethylene (5.0 μg/m^3^). All measured concentrations of carbon tetrachloride, which were relatively constant across O, S, H, and P samples, were at or near the risk threshold value (0.7 μg/m^3^). Median and 90th percentile concentrations in outdoor air were below acceptable risk limits for chloroform (0.4 μg/m^3^) and *p*-dichlorobenzene (0.9 μg/m^3^). For *p*-dichlorobenzene and chloroform, median levels in winter and 90th percentile H and P samples in both seasons exceeded the applicable reference levels. For benzene, the median (O winter only) and 90th percentile concentrations in both seasons exceeded the acceptable risk value (1.3 μg/m^3^) in O, H, and P samples. Further research is needed to better understand the significance of these results for health risk assessments of children as well as potential interventions to reduce exposure.

## Figures and Tables

**Table 1:** Sociodemographic characteristics of the 113 SHIELD households providing time–activity data and a matched H and P sample in either season.

Sociodemographic variables	Total no. (weighted %)*^a^*
School	
Whittier	60 (46.3)
Lyndale	53 (53.7)
Language	
English speaking	37 (48.0)
Non-English speaking	76 (52.0)
Race/ethnicity	
African American	24 (32.9)
Somali	27 (17.5)
Hispanic	40 (26.9)
Southeast Asian	9 (7.8)
Other*^b^*	13 (14.9)
Sex	
Male	58 (55.9)
Female	55 (44.1)
Grade	
2	27 (24.3)
3	26 (23.8)
4	32 (27.2)
5	28 (24.8)
Place of birth	
United States	62 (64.4)
Other	51 (35.6)
Household characteristics	
Smokers present	19 (21.7)
Attached garage	5 (5.5)
Use of room deodorizers	84 (73.6)
Central air conditioning	11 (8.5)
House type	
Single-family detached	32 (28.5)
Single-family attached	15 (14.6)
Apartment	64 (55.3)
Other	2 (1.7)
Rent	94 (81.0)
Own	19 (19.1)

a Totals within categories may not add to 100% due to rounding.

b Caucasian, Native American, and those indicating mixed race.

**Table 2:** Summary of VOC concentration (μg/m^3^) distributions for 15 VOCs in matched P, S, H, and O samples from 113 subjects in winter and spring 2000.

		O*^a^*				S*^b^*				H*^c^*				P*^c^*			
VOC	Season	%Det	Median	Q10	Q90	%Det	Median	Q10	Q90	%Det	Median	Q10	Q90	%Det	Median	Q10	Q90
Benzene	Winter	100.0	1.3	0.4	2.2	77.1	0.6	0.1	1.6	100.0	2.2	0.8	6.2	100.0	2.1	0.7	6.5
	Spring	100.0	1.1	0.7	1.6	90.5	0.6	0.2	1.0	99.0	2.1	0.6	7.2	100.0	1.5	0.7	4.2
Carbon tetrachloride	Winter	100.0	0.5	0.5	0.7	100.0	0.6	0.5	0.7	99.0	0.6	0.5	0.6	100.0	0.5	0.4	0.6
	Spring	100.0	0.5	0.4	0.7	93.7	0.5	0.2	0.9	100.0	0.5	0.4	0.8	100.0	0.5	0.4	0.6
Chloroform	Winter	25.0	0.1	0.1	0.1	81.3	0.2	0.1	0.3	98.0	0.8	0.3	2.6	100.0	0.7	0.3	2.1
	Spring	60.0	0.1	0.1	0.3	42.9	0.1	0.1	0.4	97.0	1.5	0.5	3.4	98.9	1.2	0.5	2.9
*p*-Dichlorobenzene	Winter	12.5	0.1	0.0	0.2	87.5	0.5	0.1	1.1	82.8	0.7	0.1	344.6	92.5	1.0	0.2	167.2
	Spring	70.0	0.2	0.1	0.4	85.7	0.5	0.1	1.1	89.9	0.9	0.2	429.0	93.2	1.3	0.2	87.2
Ethylbenzene	Winter	100.0	0.6	0.2	0.8	97.9	0.6	0.2	1.0	100.0	1.0	0.6	2.8	100.0	1.0	0.6	2.4
	Spring	100.0	0.5	0.3	0.7	93.7	0.3	0.2	0.5	100.0	1.0	0.5	3.8	100.0	0.9	0.5	2.0
*d*-Limonene	Winter	12.5	0.1	0.0	0.3	100.0	4.6	1.8	12.1	100.0	28.6	6.4	122.3	100.0	24.7	7.5	159.5
	Spring	80.0	0.4	0.1	0.6	100.0	1.9	0.9	7.9	100.0	21.2	7.2	124.8	100.0	22.2	8.3	110.0
Methylene chloride	Winter	0.0	0.3	0.2	0.6	2.1	0.4	0.1	0.6	23.2	0.4	0.2	1.3	19.4	0.4	0.2	1.3
	Spring	0.0	0.2	0.1	0.6	1.6	0.3	0.1	0.5	20.2	0.3	0.2	1.2	17.0	0.3	0.2	1.3
α-Pinene	Winter	0.0	0.0	0.0	0.1	87.5	0.2	0.1	0.3	100.0	2.4	0.7	6.5	100.0	1.9	0.7	5.1
	Spring	10.0	0.1	0.1	0.1	63.5	0.2	0.1	0.4	100.0	2.4	0.7	8.6	100.0	1.8	0.6	5.4
β-Pinene	Winter	0.0	0.1	0.1	0.1	4.2	0.1	0.1	0.1	94.9	2.5	0.5	11.7	93.5	1.7	0.4	11.5
	Spring	0.0	0.1	0.1	0.1	9.5	0.1	0.1	0.2	89.9	1.5	0.1	7.4	87.5	1.1	0.1	5.3
Styrene	Winter	0.0	0.1	0.0	0.1	31.3	0.1	0.0	0.4	91.9	0.7	0.2	1.5	93.5	0.5	0.2	1.2
	Spring	0.0	0.0	0.0	0.1	39.7	0.1	0.1	0.3	91.9	0.8	0.3	2.1	85.2	0.5	0.1	1.2
Tetrachloroethylene	Winter	75.0	0.2	0.1	0.4	95.8	0.3	0.2	0.4	98.0	0.5	0.2	1.3	100.0	0.4	0.2	1.3
	Spring	100.0	0.3	0.2	0.4	85.7	0.3	0.1	0.6	94.9	0.4	0.2	1.0	96.6	0.4	0.2	0.9
Toluene	Winter	100.0	2.6	0.9	4.2	97.9	2.9	1.4	5.6	100.0	8.2	3.5	19.2	100.0	7.7	3.4	17.7
	Spring	100.0	2.7	1.1	3.6	95.2	1.6	0.2	3.2	100.0	8.9	4.2	25.1	100.0	7.7	3.1	18.0
Trichloroethylene	Winter	62.5	0.3	0.0	1.0	72.9	0.2	0.1	0.8	82.8	0.3	0.1	0.9	90.3	0.3	0.1	0.8
	Spring	80.0	0.2	0.1	0.7	55.6	0.1	0.0	0.3	73.7	0.2	0.1	1.7	72.7	0.2	0.1	0.8
*m/p*-Xylene	Winter	100.0	2.3	0.9	3.3	100.0	2.3	1.1	3.6	100.0	3.7	2.2	10.4	100.0	3.5	2.1	8.0
	Spring	100.0	2.0	1.1	2.8	100.0	1.2	0.7	1.5	100.0	3.3	1.5	13.2	100.0	2.9	1.4	6.9
*o*-Xylene	Winter	100.0	0.8	0.3	1.1	100.0	0.8	0.3	1.2	100.0	1.2	0.7	3.2	100.0	1.1	0.7	2.6
	Spring	100.0	0.7	0.4	0.9	100.0	0.4	0.3	0.5	100.0	1.1	0.5	4.1	100.0	1.0	0.5	2.7

Abbreviations: %Det, equal to the proportion of the individual chemical concentrations above the analytical detection limit (results ≤ 0 were reset to one-half the analytical detection limit for all analyses); Q10, 10th percentile; Q90, 90th percentile.

a Five-day average; samples taken outdoors at two schools over 4 weeks in winter (*n* = 8) and 5 weeks in spring (*n* = 10).

b Five-day average; samples taken indoors in five rooms in two schools over 4 weeks in winter (*n* = 39) and 5 weeks in spring (*n* = 47), with missing results for four badges, one winter and three spring.

c H and P samples (2-day average) collected concurrently for a single child in each household (winter, *n* = 93; spring, *n* = 88). Quantiles were calculated using weights to adjust for nonselection and nonresponse.

**Table 3:** Weighted percentage of each day in each microenvironment or conducting exposure-related activities for 113 subjects during both seasons.

			Percentile				
Time spent in location/activity	Mean ± SD	Minimum	25th	50th	75th	90th	Maximum
Inside at home	65 ± 6.6	45	62.5	66	68.9	70.6	87
Inside at school	25 ± 4.4	0.5	24	25.2	26.6	28.6	40.7
Inside at other	3.2 ± 5.4	0	0	0.5	4	8.2	24.1
Outside at home	1.2 ± 2	0	0	0	1.6	4.2	7.8
Outside at school	1.3 ± 1	0	0.9	1	1.4	2.1	7
Outside at other	0.7 ± 1.3	0	0	0	1.4	2.7	7.7
In transit (traveling in vehicle)	3.6 ± 1.9	0	2.1	3.8	4.7	5.9	10.6
Indoors with smoker(s)	1.3 ± 3.8	0	0	0	0.3	4.2	22.8
In vehicle with smoker(s)	0.1 ± 0.2	0	0	0	0	0	2
Doors or windows open in residence	10.8 ± 22.4	0	0	0	6.6	49.2	100

**Table 4:** Comparison of weighted correlation coefficient (*r*^2^)for P versus H measurements and a time-weighted microenvironmental model.

VOC	Vs. H VOC concentration	Vs. time-weighted model (H, S, and O locations)
Benzene	0.50	0.51
Chloroform	0.56	0.58
*p*-Dichlorobenzene	0.80	0.83
Ethylbenzene	0.56	0.56
*d*-Limonene	0.67	0.69
Methylene chloride	0.64	0.61
α-Pinene	0.59	0.60
β-Pinene	0.39	0.41
Styrene	0.20	0.21
Tetrachloroethylene	0.59	0.58
Toluene	0.49	0.50
Trichloroethylene	0.72	0.74
*m/p*-Xylene	0.58	0.58
*o*-Xylene	0.57	0.56
